# Association of l-Arginine Supplementation with Markers of Endothelial Function in Patients with Cardiovascular or Metabolic Disorders: A Systematic Review and Meta-Analysis

**DOI:** 10.3390/nu11010015

**Published:** 2018-12-20

**Authors:** Josianne Rodrigues-Krause, Mauricio Krause, Ilanna Marques Gomes da Rocha, Daniel Umpierre, Ana Paula Trussardi Fayh

**Affiliations:** 1 School of Physical Education, Federal University of Rio Grande do Sul, Felizardo Strees 750, Porto Alegre 90690-200, RS, Brazil; josi_danca@yahoo.com.br; 2 Laboratory of Inflammation, Metabolism and Exercise Research (LAPIMEX) and Laboratory of Cellular Physiology, Department of Physiology, Institute of Basic Health Sciences, Federal University of Rio Grande do Sul, Sarmento Leite Street 750, Porto Alegre 90035-190, RS, Brazil; mauricio.krause@ufrgs.br; 3 Graduation Program in Nutrition, Federal University of Rio Grande do Norte, Senador Salgado Filho Street 3000, Natal 59078-970, RN, Brazil; ilanna.marques@gmail.com; 4 Department of Public Health, Federal University of Rio Grande do Sul, São Manuel Street S/N, Porto Alegre 90620-110, RS, Brazil; daniel.umpierre@gmail.com

**Keywords:** obesity, type 2 diabetes, cardiovascular disease, nitric oxide, flow-mediated dilation, asymmetric dimethylarginine

## Abstract

l-Arginine supplementation is a potential therapy for treating cardiovascular and metabolic diseases. However, the use of distinct l-arginine sources, intervened populations, and treatment regimens may have yielded confusion about their efficacy. This research constitutes a systematic review and meta-analysis summarizing the effects of l-arginine supplementation compared to placebo in individuals with cardiovascular disease (CVD), obesity, or diabetes. Eligibility criteria included randomized clinical trials and interventions based on oral supplementation of l-arginine with a minimum duration of three days; comparison groups consisted of individuals with the same disease condition receiving an oral placebo substance. The primary outcome was flow-mediated dilation, and secondary outcomes were nitrite/nitrate (NOx) rate and asymmetric dimethylarginine (ADMA). Statistical heterogeneity among studies included in the meta-analyses was assessed using the inconsistency index (I2). Fifty-four full-text articles from 3761 retrieved references were assessed for eligibility. After exclusions, 13 studies were included for data extraction. There was no difference in blood flow after post-ischemic hyperemia between the supplementation of l-arginine and placebo groups before and after the intervention period (standardized mean difference (SMD) = 0.30; 95% confidence intervals (CIs) = −0.85 to 1.46; I2 = 96%). Sensitivity analysis showed decreased heterogeneity when the studies that most favor arginine and placebo were removed, and positive results in favor of arginine supplementation were found (SMD = 0.59; 95% CIs = 0.10 to 1.08; I2 = 75%). No difference was found in meta-analytical estimates of NOx and ADMA responses between arginine or placebo treatments. Overall, the results indicated that oral l-arginine supplementation was not associated with improvements on selected variables in these patients (PROSPERO Registration: CRD42017077289).

## 1. Introduction

Cardiovascular (CVD) and metabolic diseases (such as obesity, insulin resistance, and diabetes) are major health problems worldwide. Such conditions are physiologically related through mechanisms that involve endocrine, nervous, and immune system cross-talk [1]. Several studies demonstrated that diabetic patients have two- to fourfold propensity to develop coronary artery disease (CAD), myocardial infarction, and other heart diseases [2]. Importantly, CVD and diabetes impose large economic burdens on the individual patient and on national healthcare systems. For this reason, the search for complementary and alternative therapies is of major interest.

Among non-pharmacological therapies, physical exercise and nutrition were extensively studied due to their potential benefits [3,4,5,6]. Particularly from the nutritional standpoint, supplementation of people with CVD and metabolic complications with amino acids [7], vitamins [8], fatty acids [9], proteins, and others [3] were used as a tool to improve immune, neural, cardiovascular, metabolic, and endocrine function. Interestingly, one of the major connections between these diseases is the availability of nitric oxide (NO), a nitrogen free radical that is continuously produced from the semi-essential amino acid, l-arginine [10]. This molecule participates in several regulatory processes such as relaxation and proliferation of vascular smooth muscle cells, angiogenesis, immune response, insulin secretion and signaling, and cell communication [1].

**Figure 1:**
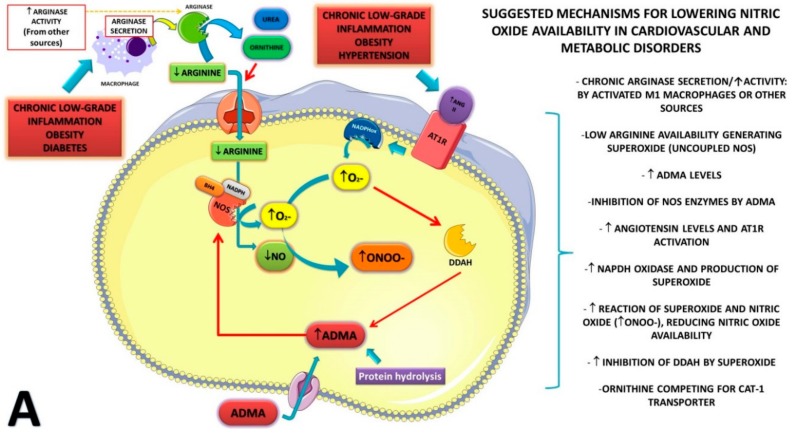

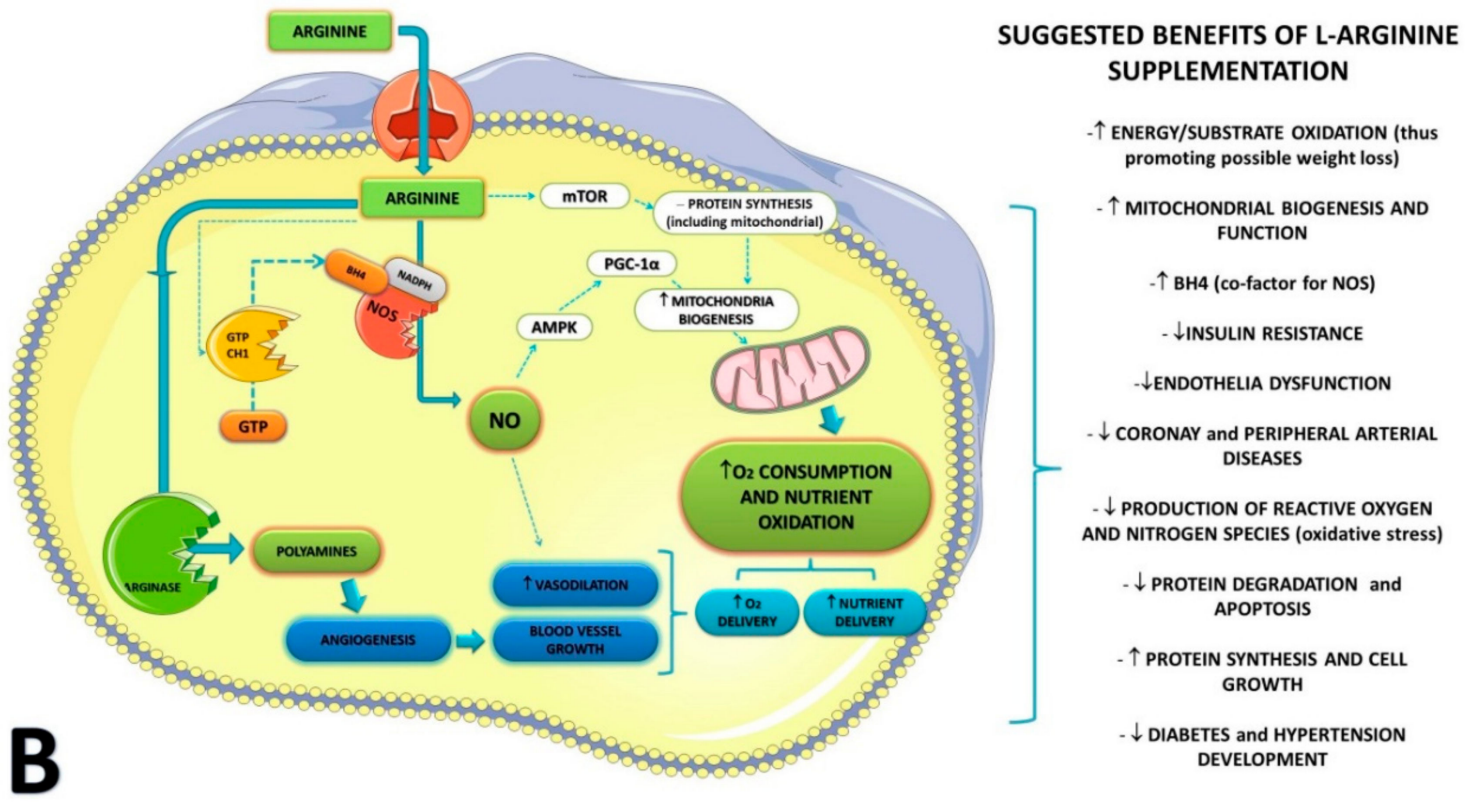
Mechanisms for lowering nitric oxide (NO) availability in cardiovascular and metabolic disorders (**A**). Increased angiotensin II (AngII) levels, asymmetric dimethylarginine (ADMA), and low plasma l-arginine concentration are all conditions likely to reduce NO· production. Inflamed adipose tissue (due to its expansion—obesity) can lead to (i) ↑ release of inflammatory cytokines; (ii) ↑ release of AngII; (iii) ↑ protein catabolism (due to the pro-inflammatory state); and (iv) ↑ activation of macrophages. Angiotensin II, acting through its receptor (AT1R), increases generation of superoxide (O^2−^), primarily through activation of reduced nicotinamide adenine dinucleotide phosphate (NAD(P)H) oxidase. O^2−^ reacts with NO∙ to form peroxynitrite (ONOO∙), a very reactive and destructive molecule, leading to decreased availability of NO∙. Moreover, superoxide is a known inhibitor of dimethylarginine dimethylhydrolase (DDAH), a key regulatory enzyme, which controls the metabolism of ADMA. ADMA is an endogenous methylated amino acid that inhibits the constitutive endothelial and neuronal isoforms of nitric oxide synthase (NOS). ADMA is released by protein hydrolysis; thus, increased catabolism induced by several inflammatory cytokines can elevate the ADMA levels. As DDAH uses ADMA as a substrate and regulates plasma levels of ADMA, it may influence the bioavailability of NO∙ and possibly contribute to changes in blood pressure. Homocysteine (an indirect substrate for the synthesis of ADMA) is also an inhibitor of the DDAH enzyme (via oxidation of a sulfhydryl group). Activation of macrophages (by pro-inflammatory cytokines) may lead to arginase secretion, an enzyme that metabolizes l-arginine to urea and l-ornithine. Chronically elevated arginase plasma levels can reduce plasma concentration and availability of l-arginine for NO∙ synthesis. l-Ornithine may also compete for the same transporter used by l-arginine (cationic amino acid transporter (CAT-1)) at the cell membrane level. In addition, low availability of l-arginine to NOS enzymes can increase superoxide synthesis via a mechanism known as endothelial NOS (eNOS) uncoupling (in the absence of sufficient l-arginine, the enzyme donates electrons to oxygen forming superoxide; however, considering the l-arginine concentrations in plasma/cells and the Michaelis constant (*K*_m_) of eNOS, the contribution of this pathway to superoxide production is still under debate). How l-arginine supplementation can aid the condition (**B**). l-Arginine supplementation can restore the levels of l-arginine and NO∙ by (i) preventing eNOS uncoupling (thus reducing superoxide formation); (ii) providing enough l-arginine for NO synthesis according to the physiological requirements; (iii) increasing guanosine triphosphate (GTP) cyclohydrolase I activity )an enzyme that is activated by l-arginine, and is the rate-limiting enzyme for the synthesis of tetrahydrobiopterin (BH4)—an essential co-factor for NOS activity). Both l-arginine and NO∙ have important metabolic functions, increasing protein synthesis pathways (by activating mammalian target of rapamycin (mTOR)) and through activation of adenosine monophosphate (AMP)-activated protein kinase (AMPK) and peroxisome proliferator-activated receptor gamma coactivator 1α (PGC-1α), lead to mitochondrial biogenesis. Metabolization of l-arginine by arginase may lead to polyamine synthesis, essential for cell growth and angiogenesis. Altogether, increased blood vessels and vasodilation (induced by normalization of NO∙ availability) will lead to increased nutrient delivery and oxygen consumption. For details on mechanisms, please read References [1,10,19]. This model describes several possibilities of mechanisms in a representative cell. Mechanisms may vary between cells due to the presence or absence of enzymes, receptors, and transporters.

Decreased production of NO∙ may result in cellular dysfunction, decreased blood flow, glucose transport, insulin resistance, insulin secretion, hypertension, and diabetes. In fact, several studies reported that NO∙ production is blunted (at the plasma or cellular environment levels) in cardiovascular and metabolic diseases, causing important physiological disturbances [11]. Among the mechanisms that underlie the lower NO∙ production/availability in metabolic and cardiovascular diseases, we can include the decreased blood levels of insulin, the increased production of angiotensin II (AngioII), hyperhomocysteinemia, increased asymmetric dimethylarginine (ADMA) synthesis, and the low plasma concentration of l-arginine [1] (Please see Figure 1A for details).

Thus, l-arginine supplementation is considered a potential therapy for the treatment of cardiovascular and metabolic diseases, targeting to normalize the NO∙ levels and other metabolites produced from l-arginine, such as polyamines (Please see Figure 1B for details). Different studies tested l-arginine for improving metabolic and cardiovascular function [12,13,14,15,16,17,18]. However, methodological variations in study populations and interventions limit the interpretability on the efficiency of l-arginine supplementation on different biomarkers of cardiovascular health or metabolic diseases.

Therefore, the aim of this systematic review and meta-analysis was to summarize randomized controlled trials (RCTs) that assessed the effects of l-arginine supplementation compared to placebo in people presenting CVD, obesity, and/or diabetes. The primary outcome was flow-mediated dilation, chosen by its properties to indicate subclinical atherosclerotic disease (in non-CVD patients), as well as vascular function when disease is already manifested. Secondary outcomes were nitric oxide metabolite formation (nitrites and nitrates, named tNOx) and ADMA. Figure 1A,B illustrate how our research question was constructed, connecting cellular mediators (NOx and ADMA) of l-arginine synthesis to a clinical outcome (blood flow), and the rationale for using l-arginine supplementation.

## 2. Methods

This review was conducted following the Cochrane Handbook for Systematic Reviews of Interventions (Collaboration 2011) and reporting adheres to the Preferred Reporting Items for Systematic Reviews and Meta-Analysis (PRISMA) guidelines [20] This study was registered at PROSPERO international prospective register of systematic reviews (CRD42017077289).

### 2.1. Search Strategy and Study Selection

Eligible studies were identified through a systematic search without language restrictions of the following electronic databases, from the earliest to 21 July 2017: Medical Literature Analysis and Retrieval System Online MEDLINE (accessed by Pubmed), Excerpta Medica dataBASE (Embase), Cochrane Wiley (Central Register of Controlled Trials), ClinicalTrials.gov, Physiotherapy Evidence Database (PEDRO), and “*Literatura Latino-Americana e do Caribe em Ciências da Saúde*” (LILACS). We additionally carried out manual searches in reference lists of selected published studies.

A combination of terms was used to identify relevant publications: (1) cardiovascular disease, arginine, and vascular or insulin sensitivity outcomes, and (2) obesity, arginine, and vascular or insulin sensitivity outcomes. The exploded versions of the Medical Subject Headings (MeSH) were (1) (Heart Diseases OR Coronary Disease Coronary OR Heart Disease OR Coronary Artery Disease OR Myocardial Infarction OR Myocardial Revascularization), and (2) (Obesity OR Overweight OR Abdominal Obesity OR Visceral Obesity OR Central Obesity OR Obese OR excess body weight) AND (Arginine OR l-Arginine OR Arginine*) AND (Endothelium OR endothelial OR vascular OR “intima-media” OR “brachial artery” OR “flow mediated dilation” OR “flow mediated dilatation” OR “fmd” OR “hyperemia” OR vasomotor OR vasodilation OR vasodilatory) OR (Insulin Resistance OR Insulin sensitivity OR HOMA OR Clamp OR Glucose tolerance OR Glucose challenge). The search strategy was peer-formulated by an information specialist (D.U.).

### 2.2. Eligibility Criteria

We included RCTs that compared arginine supplementation with a placebo control group in subjects with any type of cardiovascular disease (coronary artery disease, peripheral artery disease, chronic heart failure, myocardium infarction, angina, etc.), obesity (mean body mass index (BMI) >30 kg/m^2^, waist circumference >88 cm), and/or type 2 diabetes mellitus (T2DM; glycated hemoglobin (HbA1c) ≥6.5%; fasting glycaemia ≥126 mg/dL; glycemia 2 h glucose tolerance test (GTT) ≥200 mg/dL).

The interventions included oral supplementation of l-arginine (capsule, biscuit, barre, shake, syrup, etc.) with a minimum duration of three days, but no limit set for maximum duration or dose of l-arginine supplementation. The comparisons included a control group with the same health condition, receiving an oral supplementation of a placebo substance for the same duration as the arginine supplementation.

Exclusion criteria included healthy patients, intravenous arginine administration, combined interventions (l-arginine plus other active substances, or diet/exercise associated), placebo groups with an active component, a duration of the intervention shorter than three days, non-randomized controlled trials, and studies without outcomes of interest. To better standardize the units of measurement for later data extraction and unit transformation, studies including outcomes of interest, but with different measurement techniques or body regions of assessment (i.e., plethysmography) were excluded.

The main clinical outcome of this review was blood flow after post-ischemic hyperemia, assessed by flow-mediated dilation (FMD), using the ultrasound technique. Secondary biochemical outcomes were nitrite/nitrate (NOx) rate and asymmetric dimethylarginine (ADMA), assessed by known enzymatic colorimetric assays. Data were extracted as means or differences between means and dispersion values at the onset (baseline) and at the end of interventions. Articles in English, Portuguese, or Spanish were considered for review.

### 2.3. Data Extraction

Two independent investigators (J.R-K. and A.P.T.F.) evaluated titles and abstracts of retrieved articles. Abstracts that did not provide enough information about eligible reasons for exclusion were left for the full-evaluation stage. The same reviewers independently assessed full-text articles, defining eligible studies, and continued the flow work for data extraction. Disagreements were solved by consensus or by a third reviewer (I.M.G.R). Specific characteristics of intervention (duration of the intervention, dose of l-arginine, type of oral administration, etc.), age and gender of participants, clinical condition, specific outcomes and methods of assessments, adherence, and/or dropout rates were also extracted.

### 2.4. Risk of Bias Assessment

Risk of bias of individual studies was evaluated according to the Cochrane Collaboration tool for assessing risk of bias in randomized trials [21], a seven-item instrument, as follows: (1) adequate sequence generation; (2) allocation concealment; (3) blinding of participants and personnel; (4) blinding of outcomes assessors; (5) incomplete outcome data; (6) selective reporting; and (7) other bias. Risk of bias of each of the seven domains was expressed in the format “low”, “high”, or “unclear” risk. The standards of assessment for risk of bias (by consensus of the authors) were as follows: low risk of bias detected for seven items = “low” risk of bias, unclear risk for one to two items = “some” risk of bias, unclear risk for more than three items = “high” risk of bias.

### 2.5. Data Analysis

Estimates of pooled effects were obtained by comparing absolute post-intervention means for each group for NOx and ADMA outcomes (μmol/L), and standardized mean differences (SMD) for blood flow outcome. Results were expressed as means ± standard deviation (SD) between the groups arginine vs. placebo. Unit transformation for post-intervention mean values and respective dispersion values were performed when appropriate, in accordance with Cochrane Collaboration recommendations (Collaboration 2011). Calculations were performed using a random-effect model. An α value = 0.05 was considered statistically significant.

The inconsistency index (I2) was used to quantify statistical heterogeneity in meta-analyses, and values greater than 50% were considered indicative of high heterogeneity [21]. Heterogeneity was explored as follows: (i) by carrying out additional meta-analysis removing each study at a time to check if a particular study was solely affecting heterogeneity; and (ii) conducting a sensitivity analysis based on a review to evaluate previous relevant clinical information. All analyses were conducted using Reviewer Manager Software version 5.3.

## 3. Results

### 3.1. Study Selection Process

From 3743 potentially relevant citations identified in our structured searches and reference lists (18 citations), 1990 records were screened (after removing duplicates) and 54 full-text articles were assessed for eligibility. Next, 41 articles were excluded due to the following reasons: type of patient (with no cardiovascular disease, obesity, or T2DM), comparison group (other type of substances rather than placebo), non-specific outcomes or methods of assessment, type of intervention (arginine combined with other substances), study design (non-randomized trials), language (two articles in Korean), multiple publications (data of different outcomes, but with the same set of participants and intervention, published in two different articles of the same research team), and lack of author responses (failed attempts to get lacking numerical data from the published article).

**Figure 2:**
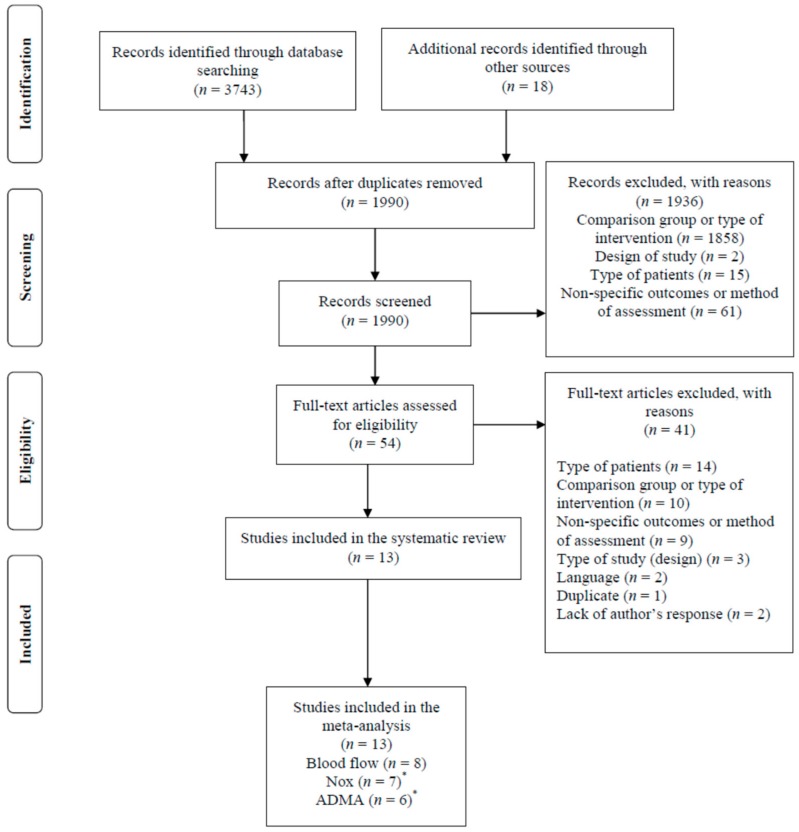
Flow diagram of search and selection of studies. ^*^Nox and ADMA: Results of Schneider et al (2015) included two separated studies with two different types of patients: Coronary Artery Disease (CAD) and Peripheral Artery Disease (PAOD).

Finally, 13 studies were included in the systematic review and meta-analysis. Specifically, eight studies were used for the blood flow meta-analysis [12,13,14,15,16,17,18,22], seven for NOx [13,23,24,25,26], and six for ADMA [15,22,25,26,27] outcomes. The study by Schneider et al. [26] included two separated sets of results, with two different types of patients: coronary artery disease (CAD) and peripheral artery occlusive disease (PAOD). This study was used for NOx and ADMA meta-analyses, and its sub-studies are referred to as “Schneider 2015 CAD” and “Schneider 2015 PAOD”, respectively. Figure 2 shows a flow diagram of the study selection process. Table 1 shows the characteristics of these studies. Additionally, Table S1 (Supplementary Materials) is available with information about full-text articles and reasons for exclusion in the meta-analysis.

### 3.2. Characteristics of Participants

Included studies in the meta-analyses had a total of 723 participants involved with the interventions (358 participants receiving arginine treatment) or control groups (365 participants receiving placebo treatment) [12,13,14,15,16,17,18,22,23,25,26,27,28]. Of these, two studies included obese and/or T2DM patients (pooled *N* = 280, with 139 and 141 patients for arginine and placebo treatment, respectively) [23,25,28]. Cardiovascular conditions were present in 443 participants (219 and 224 for arginine and placebo treatments, respectively). Specifically, there were 219 patients with coronary artery disease [13,14,15,16,26], 136 with peripheral artery disease [18,22,26], 30 with heart failure [18], 38 with angina [27], and 20 with arteriosclerosis [12]. The age range of participants was between 33 and 73 years old, including both male and female participants, although the male gender was predominant in 10 studies [12,13,14,15,16,17,22,25,26,27]. Table 1 shows the descriptive characteristics of the included studies.

### 3.3. Characteristics of Interventions

l-Arginine or placebo treatments were orally administrated in all included studies via tablets or capsules [13,14,22,23,25,26,28], bars [16], or powder [12], and three studies did not report the form of administration [15,17,27]. The duration of the treatments ranged from the shortest being three days [12,18] to the longest of 18 months [17], with six months being the most often used duration [15,22,25,26,28]. The minimum arginine dose offered was 1.2 g/day [25], and the maximum was 15 g/day [27], while 6.4 [15,16,17] and 9.0 [13,14,28] g/day were the most often used doses.

Adherence rates were reported in six studies [12,16,22,23,25,27], at around 80% for most of the studies. Adverse events were reported in five studies, such as transient skin dermatitis and dysmenorrhea [23], dizziness [17], nausea, stomach cramps [13,22], and diarrhea [26]. No adverse effects were noted during the treatment in three studies [12,15,25].

### 3.4. Risk of Bias

From the 13 assessed studies, risk of bias of individual studies was classified as “low” risk (none or only one item “unclear”) in three studies [15,23,26], “some” risk (up to two items “unclear”) in six [12,14,16,17,18,25,27,28], and “high” risk (more than two items “unclear”) in another two studies [13,22]. Overall, the major weaknesses among studies were the lack of clarity regarding concealment of the allocation sequence, blinding of participants, and outcome assessment. Table 2 shows the risk of bias of individual studies.

### 3.5. Effect Measures

#### 3.5.1. Blood Flow

**Figure 3:**
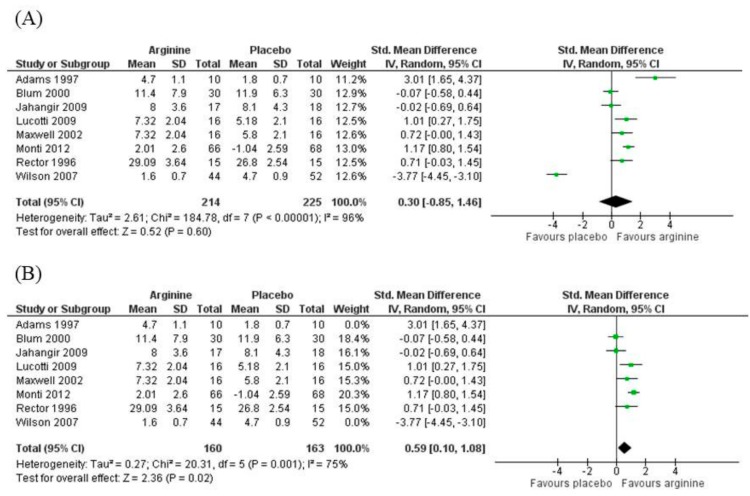
(**A**) Blood flow responses of individual studies: l-arginine vs. placebo treatment. (**B**) Sensitivity analysis of blood flow responses of individual studies: l-arginine vs. placebo treatment (removing the studies that most favor arginine and placebo). SD = standard deviation; CI = confidence interval.

Considering the eight studies included in the meta-analysis of the blood flow after post-ischemic hyperemia [12,13,14,15,16,17,18,22], there was no difference found between the supplementation of arginine and placebo groups before and after the intervention period (SMD = 0.30; 95% confidence intervals (CIs) = −0.85 to 1.46; *N* = 469, *Z* = 0.52 (*p* = 0.60), P for heterogeneity <0.001, I2 = 96%) (Figure 3A). However, a sensitivity analysis showed decreased heterogeneity when the studies that most favor arginine [12] and placebo [22] were removed, and positive results in favor of arginine supplementation were found (SMD = 0.59; 95% CIs = 0.10 to 1.08; *N* = 323, *Z* = 2.36 (*p* = 0.02), P for heterogeneity <0.001, I2 = 75%) (Figure 3B). Removing the only study that included obese patients for the analysis of blood flow [17], no change in heterogeneity was observed, favoring neither l-arginine nor placebo interventions.

#### 3.5.2. Nitrites/Nitrates (NOx)

**Figure 4:**
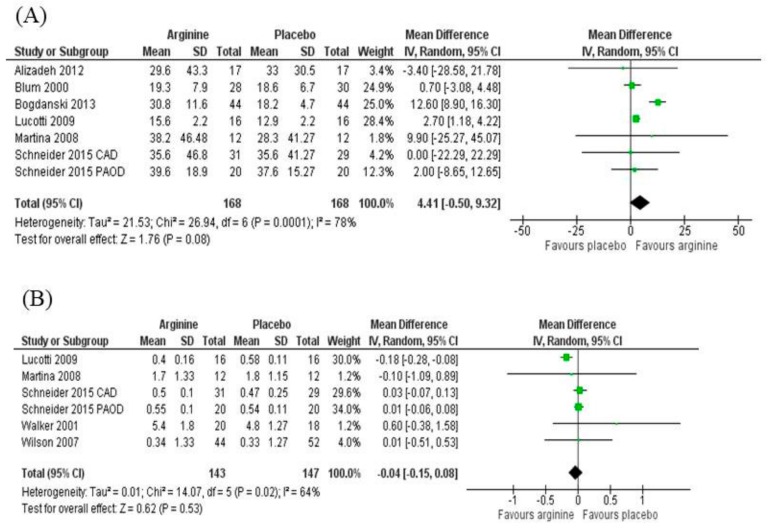
(**A**) NOx and (**B**) ADMA responses of individual studies: l-arginine vs. placebo treatment.

Taking into account all types of patients in the meta-analysis [13,15,17,23,26,28], no difference was found in the mean difference of NOx responses between arginine or placebo treatments (4.41 μmol/L; 95% CIs = −0.50 to 9.32; *N* = 336, *Z* = 1.76 (*p* = 0.08), P for heterogeneity <0.001, I2 = 78%) (Figure 4). On the other hand, a subgroup analysis showed that, upon only considering obese/T2DM patients or cardiovascular disease patients in separate subgroups, there was a positive polled effect of arginine supplementation on NOx response: obese [23,25,28]: 12.24 μmol/L; 95% CIs = 8.60 to 15.88; *N* = 146, *Z* = 6.59 (*p* < 0.001), P for heterogeneity = 0.46, I2 = 0%; cardiovascular disease ([13,26]: 2.40 μmol/L; 95% CIs = 1.00 to 3.80; *N* = 190, *Z* = 3.37 (*p* = 0.0008), P for heterogeneity = 0.81, I2 = 0%.

#### 3.5.3. Asymmetric Dimethylarginine (ADMA)

No differences were found for the mean difference of ADMA responses comparing arginine and placebo treatments (−0.04 μmol/L; 95% CIs = −0.15 to 0.08; *N* = 290, *Z* = 0.62 (*p* = 0.53), P for heterogeneity = 0.02, I2 = 64%) (Figure 4). A sensitivity analysis showed no heterogeneity when removing the study of Lucotti [15], although it did not alter the final effect in favor of arginine or placebo (0.02 μmol/L; 95% CIs = −0.04 to 0.07; *N* = 258, *Z* = 0.64 (*p* = 0.52), P for heterogeneity = 0.82, I2 = 0%). Removing the only study not evaluating patients with cardiovascular conditions [25] did not influence heterogeneity or the final pooled effect.

## 4. Discussion

This systematic review with meta-analysis aimed to verify the effects of l-arginine supplementation compared to placebo on blood flow, NOx, and ADMA responses in people with CVD, obesity, and/or diabetes using RCTs. We firstly demonstrated that there was no difference in blood flow responses comparing l-arginine with placebo supplementation in patients with cardiovascular and/or metabolic disorders. However, under sensitivity analysis, the exclusion of studies with extreme responses favoring arginine [12] or placebo [22] suggested superiority of arginine supplementation regarding blood flow improvements. Secondly, there was no difference in NOx and ADMA responses comparing arginine with placebo supplementation in these patients, although speculation on subgroup responses indicates that obese/T2DM patients could improve NOx response and further endothelial function as a result of arginine supplementation. Although we pre-specified subgroup analyses, we underscored that (i) NOx and ADMA subgroup meta-analyses were based on few studies, and (ii) distinct sample sets of individuals precluded us from exploring subgroup estimates of blood flow.

Recently, a few meta-analyses were published on oral l-arginine supplementations in patients with cardiovascular disease. In a meta-analysis of randomized controlled trials assessing the effect of l-arginine supplementation on clinical outcomes (all-cause mortality, myocardial reinfarction, successful resuscitation, shock/pulmonary edema, recurrent myocardial ischemia, and hospitalization for heart failure) in patients with acute myocardial infarction, interventions (both providing 9 g of l-arginine per day orally) were not associated with significant change in the risk of total events [29]. Neither of the two studies included in this previous review were included in our study due to the absence of outcomes of interest. Another meta-analysis aimed to examine the effect of oral l-arginine supplementation on blood pressure. Dong et al. summarized 11 randomized controlled trials with oral l-arginine interventions with dosages from 4 to 24 g/day [30]. Compared with placebo, l-arginine supplementation significantly lowered systolic blood pressure by 5.39 mm Hg (95% CIs = −8.54 to −2.25) and diastolic blood pressure by 2.66 mm Hg (95% CIs = −3.77 to −1.54). Sensitivity analyses for studies with a minimum duration of four weeks and studies in which participants did not use antihypertensive medications yielded similar results. These discrepant results and different populations demonstrate that the effects of l-arginine supplementation are not yet fully understood in patients with cardiovascular disease.

However, even though oral l-arginine supplementation seems to be a plausible strategy for improving endothelial dysfunction in patients with coronary risk, primary studies showed mixed results [12,13,22]. Such high heterogeneity is a common characteristic of meta-analyses that involve chronic interventions, such as diet or supplementation, and may be influenced by a number of factors such as characteristics of patients, dosage of substances, types of medical treatment, and sample size [21]. In the present study, we could only observe positive effects of arginine supplementation on blood flow responses when removing the more heterogeneous studies of the meta-analysis.

In this regard, Adams et al. [12] evaluated a small number of individuals (*n* = 10); thus, the statistical power (not reported) may have been low to provide estimate differences. Also, Wilson et al. [22] did not show positive effects of l-arginine supplementation (3 g/day) during six months on blood flow responses, possibly because larger doses of 5–15 g/day would be required to improve endothelial function in humans [31]. In fact, the average daily consumption of l-arginine in the American diet is 5.4 g, although human studies increased the oral intake of l-arginine supplementation from two to five times. In addition, the study by Wilson included patients with several cardiovascular risk factors, such as diabetes and hypertension, which may cause both intra- and inter-study heterogeneity.

In addition to sample size and the arginine dose, it should be considered that vasodilation is mediated by several mechanisms (independent of l-arginine availability) and molecules (potassium, oxygen, purines, and prostaglandins), and not only by NO [32]. This may also be considered when interpreting a non-significant meta-analytical estimate of the effects of l-arginine supplementation on blood flow. In this regard, we point out that trials herein summarized often evaluated physiological or clinical outcomes without assessing whether the expected biochemical effect was triggered (increase in bioactive NO). Therefore, there is uncertainty if l-arginine supplementation yields any influence on blood flow or endothelial function even when increases in bioactive NO are achieved.

Regarding NOx and ADMA responses, there were no changes overall in comparing l-arginine with placebo supplementation in the selected group of patients taken together. Large confidence intervals indicate high heterogeneity in the individual studies alone and among studies, which may influence polled results. In addition, biochemical markers have great variability depending on the measurement technique, time of the day, and type of patients, etc. [33]. For example, a recent meta-analysis showed that ELISA measurements overestimated plasma levels of ADMA compared to high-performance liquid chromatography (HPLC) [34,35].

On the other hand, in performing a subgroup analysis for type of patients, we found that, by only considering obese/T2DM patients or cardiovascular disease patients in separate subgroups, there was a positive polled effect of arginine supplementation on NOx response. For example, Alizadeh et al. [23] and Bogdanski et al. [28] found that l-arginine supplementation increased NOx levels in obese people, and Martina [25] found the same result in T2DM patients. The same occurred in only analyzing the patients with cardiovascular diseases [13,15,26]. This particular positive response may be associated with the fact that l-arginine, along with other amino acids (l-arginine precursors), such as l-glutamine, are chronically decreased in the plasma of insulin-resistant/obese people [36,37]. Thus, correction of the l-arginine availability may restore NOx (NO production), but supplementation above the physiological levels may not induce any further NO increase.

Even though the analysis of very few studies may limit conclusions, it supports a physiological speculation on possible mechanisms that may be particularly involved in the effects of arginine supplementation on endothelial function of patients with metabolic or cardiovascular disorders. In comparing ADMA and NO metabolism (Figure 1A), NO production can be affected by several signaling molecules, including ADMA, insulin sensitivity, level of inflammation, and l-arginine availability, while ADMA levels are mainly controlled by dimethylarginine dimethylhydrolase (DDAH) activity and protein hydrolysis [1]. Thus, restoration of l-arginine availability through supplementation may directly induce NO production, which may explain why NOx response to l-arginine supplementation and ADMA did not change in the included studies. Changes in ADMA levels may require longer periods of the disease to take place (thus, longer exposure to oxidative stress damage), and to interfere in NO metabolism and vasodilation. Thus, it is unlikely to reduce with short periods of l-arginine supplementation.

Hypercholesterolemia is also known to increase ADMA, but endothelial dysfunction in the setting of hypertension or diabetes is not accompanied by increased ADMA. Thus, the clinical and mechanistic data suggest that subjects with hypercholesterolemia may be more likely to benefit from l-arginine than normal subjects or those with other forms of vascular disease [38]. In the present meta-analysis, the disease does not appear to influence the ADMA response to l-arginine supplementation. Although ADMA appears to be a potential mediator of oxidative stress, the association between higher levels of ADMA and increased cardiovascular risk is still unclear.

The results of this meta-analysis should be interpreted with caution due to some limitations. Firstly, we included studies with individuals with different cardiovascular risk factors, resulting in a heterogeneous sample. Secondly, the sample sizes of individual trials were small, which might more easily suffer from sample imbalances and an influence of baseline confounding factors. Thirdly, the validity of the present meta-analysis depended upon the quality of the individual studies. It should also be considered that risk of bias in individual studies was detected. Although all studies were randomized and placebo-controlled trials, allocation concealment, quality of randomization, and details of blinding were not always reported (See Table 2). Moreover, a large variation in the supplementation duration was observed among the studies (three days to 18 months), which may impact adverse effects and safety (reported in very few studies) of l-arginine supplementation. Fourthly, the small number of limited studies limits us in the analysis of publication bias, as we planned and stated in this synthesis registration. Fifthly, varied sample groups could include patients who were likely taking medications that would impact the primary and secondary outcomes; however, we were unable to assess whether this may have happened. Lastly, the studies usually assess a clinical outcome without checking if the expected biochemical effect actually happened, and limitations can exist in interpreting if l-arginine is able to change the outcomes even when bioactive NO is increased.

Finally, although the inclusion of studies with individuals presenting different cardiovascular risk factors and undergoing different supplementation strategies may result in high heterogeneity, to the best of our knowledge, this is the first review that systematically looked at the overview of arginine supplementation on clinical (blood flow) and biochemical markers (NOx and ADMA) of endothelial dysfunction in people experiencing cardiovascular risk. We might also speculate that the benefits of the intervention may outweigh the risks when subgroups of patients are analyzed, at least regarding the mechanisms involving NOx responses related to improvements in blood flow and endothelium function. Future research should focus on the development of higher-quality RCTs in specific subgroups of patients, and comparison with other types of intervention and supplementation duration. Also, analyzing a number of other biomarkers would clarify how l-arginine may induce or not induce positive effects on endothelial function and associated cardiovascular risk.

## 5. Conclusions

Overall, this systematic review and meta-analysis of randomized, double-blind, placebo-controlled studies indicates that oral l-arginine supplementation does not induce improvements on blood flow and biochemical markers (NOx and ADMA) of endothelial dysfunction in patients with cardiovascular and/or metabolic disorders. However, removing studies that increased heterogeneity level indicated that l-arginine supplementation may have a positive impact on blood flow responses, and perhaps could be used as a strategy to improve endothelial function in the selected group of patients. NOx responses seem to be particularly influenced by the type of patient, showing positive effects of arginine supplementation when obese and/or T2DM or patients with cardiovascular diseases are analyzed separately. However, further research should be performed in order to strengthen this speculation. l-Arginine supplementation does not seem to have influence on ADMA responses. Considering the high heterogeneity levels, mainly due to different types of participants and intervention strategies, the level of evidence generated by this systematic review may not be sufficient, but it does provide an insight into how arginine supplementation may be a potential strategy to improve endothelial function in people experiencing cardiovascular risk.

## Figures and Tables

**Table 1:** Characteristics of studies verifying the effects of oral supplementation of l-arginine compared to placebo treatments on blood flow, nitrate/nitrite (NOx), and asymmetric dimethylarginine (ADMA) responses in patients with obesity, type 2 diabetes mellitus, and cardiovascular diseases.

Reference and Type of Study	Condition and Baseline Characteristics	Supplementation and Dosage		Duration of Supplementation	Outcomes Measured/ Analysis Method	Conclusions
		Arginine Group	Placebo Group			
Patients with Obesity						
Alizadeh et al. [23] Prospective, randomized, double-blind, placebo-controlled trial	Premenopausal women with central obesity (*n* = 87).	*n* = 17 33.6 ± 8.6 years Hypocaloric diet enriched in legumes (HDEL) + l-arginine (5 g/day) in form of tablets	*n* = 17 33.8 ± 9.1 years Hypocaloric diet enriched in legumes (HDEL) + placebo (starch and lactose in form of tablets)	Six weeks	(1) NOx/Griess reaction	HDEL + placebo increased NOx levels, but adding l-arginine eliminated the beneficial effect of HDEL.
Bogdanski et al. [28] Prospective, randomized, double-blind, placebo-controlled trial	Obese patients (*n* = 88)	*n* = 44 (21 male) 43.1 ± 8.6 years l-arginine (9 g/day) in form of capsules	*n* = 44 (24 male) 41.5 ± 9.1 years Placebo in form of capsules	Six months	(1) NOx/enzyme immunoassay (ELISA)	Treatment with l-arginine resulted in significant increase in NOx. No significant changes between analyzed variables were noticed in placebo group.
Monti et al. [17] Prospective, randomized, double-blind, placebo-controlled trial	Patients with impaired glucose tolerance and metabolic syndrome (*n* = 144)	*n* = 72 (42 male) 57.2 ± 11.7 years l-arginine (6.4 g/day)	*n* = 72 (39 male) 58.2 ± 9.4 years Placebo	18 months	(1) Blood flow/venous occlusion plethysmography of the forearm artery	Treatment with l-arginine increased post-ischaemic hyperemia, and no changes were observed in placebo group.
Patients with Type 2 Diabetes						
Martina et al. [25] Prospective, randomized, double-blind, placebo-controlled trial	Male patients with type 2 diabetes and hypertension (*n* = 24)	*n* = 12 62.5 (59.3–74.5) years 600 mg *N*-acetylcysteine (NAC), one tablet twice a day + l-arginine (1,200 mg/day) one vial	*n* = 12 67.0 (51.0–69.7) years Placebo in form of compounds identical in appearance to NAC and l-arginine	Six months	(1) NOx/ Griess reaction. (2) Blood flow/ultrasound for assessment the endothelial- dependent flow-mediated vasodilation of the brachial artery	In comparison with baseline, l-arginine + NAC reduced intima-media thickness and increase plasma nitrites and nitrates.
Patients with Cardiovascular Disease						
Adams et al. [12] Prospective, randomized crossover, double-blind, placebo-controlled trial	Men with angiographically documented coronary artery disease in at least two vessels (*n* = 10)	*n* = 10 41 ± 2 years l-arginine (7 g/day) in form of powder	The same group (crossover design) Placebo in form of powder with same flavor and appearance	Three days	(1) Blood flow/ultrasound (brachial artery for assessment the endothelial- dependent (reactive hyperemia) and independent (response to glyceryltrinitrate) flow-mediated vasodilation	Treatment with l-arginine improved endothelium-dependent dilatation, but no changes were seen in endothelium-independent dilatation of the brachial artery.
Maxwell et al. [16] Prospective, randomized crossover, double-blind, placebo-controlled trial	Patients with angina secondary to atherosclerotic coronary artery disease (*n* = 36)	*n* = 36 (28 male) 65.9 ± 10 years. l-arginine (6.6 g/day) in form of two bars with 3.3 g each	The same group (crossover design) Placebo bar with the same weight, appearance and flavor.	Two weeks	(1) Blood flow/ultrasound for assessment the endothelial- dependent flow-mediated vasodilation of the brachial artery	Treatment with l-arginine improved flow-mediated vasodilation.
Jahangir et al. [14] Prospective, randomized, double-blind, placebo-controlled trial	Patients with coronary artery disease (*n* = 109)	*n* = 26 (23 male) 60 ± 9 years l-arginine (9 g/day) in form of tablets	*n* = 26 (22 male) 58 ± 12 years Placebo (lactose) in form of tablets	Four days	(1) Blood flow/ultrasound (brachial artery for assessment the endothelial- dependent (reactive hyperemia) and independent (response to glyceryltrinitrate) flow-mediated vasodilation	Treatment with l-arginine had no effects on vascular function.
Lucotti et al. [15] Prospective, randomized, double-blind, placebo-controlled trial	Patients with cardiovascular disease, nondiabetic, previously submitted to an aortocoronary bypass (*n* = 30)	*n* = 16 (15 male) 65 ± 10 years l-arginine (6.4 g/day)	*n* = 14 (13 male) 64 ± 11 years Placebo with similar appearance of l-arginine	Six months	(1) NOx/Griess reaction (2) Asymmetric dimethylarginine (ADMA)/high-performance liquid chromatography (3) Blood Flow/ultrasound (brachial artery for assessment the endothelial-dependent (reactive hyperemia) vasodilation	Compared with placebo, l-arginine decreased NOx and ADMA levels, but no changes in basal blood flow were observed.
Blum et al. [13] Prospective, randomized crossover, double-blind, placebo-controlled trial	Patients with coronary artery disease (*n* = 30)	*n* = 30 (29 men) 67 ± 8 years l-arginine (9 g/d) in form of capsules	The same group (crossover design) Placebo with capsules identical to l-arginine	One month	(1) NOx/chemiluminescent technique (2) Blood flow/ultrasound (brachial artery for assessment the endothelial- dependent (reactive hyperemia) and independent (response to glyceryltrinitrate) flow-mediated vasodilation	No effects were observed on NOx and on brachial artery diameters, flow-mediated dilation, or nitroglycerin-induced dilation.
Rector et al. [18] Prospective, randomized crossover, double-blind, placebo-controlled trial	Patients with heart failure (*n* = 15)	*n* = 15 (14 male) 56 ± 10 years l-arginine (5.6 g/day (*n* = 9) or 12.6 g/day (*n* = 6)	The same group (crossover design) placebo capsules	Six weeks	(1) Blood flow/venous occlusion plethysmography	Treatment with l-arginine did not change forearm blood flow.
Schneider et al. [26] Prospective, randomized, double-blind, placebo-controlled trial	Patients suffering from peripheral arterial occlusive disease	*n* = 20 (16 male) 67.3 ± 8.0 years l-arginine (9.96 g/day) in form of effervescent tablets	*n* = 20 (15 male) 68.4 ± 8.0 years Placebo in form of tablets	Three months	(1) ADMA, nitrites, and nitrates (plasma and urine)/validated with mass spectrometry-based methods	Treatment with l-arginine increased insignificantly the ADMA concentration in the plasma, but enhanced the excretion rate of ADMA.
Schneider et al. [26] Prospective, randomized, double-blind, placebo-controlled trial	Patients suffering from coronary artery disease	*n* = 31 (24 male) (62 years—no standard deviation was informed) l-arginine (9.96 g/day) in form of effervescent tablets	*n* = 29 (24 male) (62 years—no standard deviation was informed) Placebo in form of tablets	Six months	(1) ADMA, nitrites, and nitrates (plasma and urine)/validated with mass spectrometry-based methods	Compared to placebo, plasma ADMA, nitrite, and nitrate did not change significantly with oral l-arginine supplementation. Urinary ADMA increased only marginally after three but not after six months of l-arginine supplementation. Urinary excretion of nitrate and nitrite did not significantly change after l-arginine supplementation for 3 and 6 months.
Walker et al. [27] Prospective, randomized, double-blind, placebo-controlled trial	Men with stable angina (*n* = 40)	*n* = 21 60 ± 2 years l-arginine (15 g/day)	*n* = 19 63 ± 2 years Placebo (lactose)	Two weeks	(1) ADMA/high-performance liquid chromatography (2) Blood flow/venous occlusion plethysmography of the forearm artery	Treatment with l-arginine supplementations did not alter plasma ADMA, and did not improve endothelium dependent vasodilation.
Wilson et al. [22] Prospective, randomized, double-blind, placebo-controlled trial	Patients with intermittent claudication due to peripheral arterial disease (*n* = 133)	*n* = 66 (48 male) 73 ± 9 years l-arginine (3 g/day) in the form of capsules	*n* = 67 (53 male) 72 ± 7 years Placebo in the form of capsules	Six months	(1) NOx/Griess reaction (2) ADMA/immunoassay (3) Blood flow/ultrasound (brachial artery for assessment the endothelial- dependent (reactive hyperemia) and independent (response to glyceryltrinitrate) flow-mediated vasodilation	Treatment with l-arginine did not increase nitric oxide synthesis or improve vascular reactivity.

**Table 2:** Risk of bias of individual studies.

Study	Risk of Bias						
	Random Sequence Generation	Allocation Concealment	Selective Reporting	Other Bias	Blinding of Participants, Personnel	Blinding of Outcome Assessment	Incomplete Outcome Data
Schneider et al. [26]	Low	Low	Low	Low	Low	Low	Low
Bogdanski et al. [28]	Low	Low	Low	Low	Unclear	Low	Low
Monti et al. [17]	Low	Low	Low	Low	Unclear	Low	Low
Alizadeh et al. [23]	Low	Low	Low	Low	Low	Low	Low
Jahangir et al. [14]	Unclear	Low	Low	Low	Low	Low	Low
Lucotti et al. [15]	Low	Low	Low	Low	Low	Low	Low
Martina et al. [25]	Low	Low	Low	Low	Unclear	Low	Low
Wilson et al. [22]	Unclear	Unclear	Low	Low	Low	Low	Low
Maxwell et al. [16]	Unclear	Low	Low	Low	Unclear	Low	Low
Walker et al. [27]	Unclear	Unclear	Low	Low	Low	Unclear	Low
Blum et al. [13]	Unclear	Low	Low	Low	Unclear	Unclear	Low
Rector et al. [18]	Low	Low	Low	Low	Unclear	Unclear	Low
Adams et al. [12]	Unclear	Low	Low	Unclear	Low	Unclear	Low

## Supplementary Materials

The following are available online at http://www.mdpi.com/2072-6643/11/1/15/s1.Table S1. Full text articles and reasons of exclusion in the metanalysis.
