# Supplementary material for: Association of l-Arginine Supplementation with Markers of Endothelial Function in Patients with Cardiovascular or Metabolic Disorders: A Systematic Review and Meta-Analysis
Source: Nutrients. 2018 Dec 20;11(1):15. doi: 10.3390/nu11010015 (PMC6357192; doi:10.3390/nu11010015)
Supplement: Supplementary file 1 [file nutrients-11-00015-s001.pdf]

Table S1. Full text articles and reasons of exclusion in the metanalysis

Reference	Status	Reason of Exclusion
Schneider et al. Effects of chronic oral L-arginine administration on the L-arginine/NO pathway in patients with peripheral arterial occlusive disease or coronary artery disease:L-Arginine prevents renal loss of nitrite, the major NO reservoir. Amino Acids. 2015,47(9):1961-74.	Included	--
Bogdanski et al. Supplementation with L-arginine favorably influences plasminogen activator inhibitor type 1 concentration in obese patients. A randomized, double blind trial. J Endocrinol Invest. 2013. 36(4): 221-6.	Included	--
Monti et al . Effect of a long-term oral l-arginine supplementation on glucose metabolism: a randomized, double-blind, placebo-controlled trial. Diabetes Obes Metab. 2012, 14(10):893-900.	Included	--
Alizadeh et al. Effect of L-arginine and selenium added to a hypocaloric diet enriched with legumes on cardiovascular disease risk factors in women with central obesity: a randomized, double-blind, placebo-controlled trial. Ann Nutr Metab. 2012. 60(2): 157-68	Included	--
Jahangir et al. The effect of L-arginine and creatine on vascular function and homocysteine metabolism. Vasc Med. 2009, 14(3):239-48.	Included	--
Lucotti et al. Oral L-arginine supplementation improves endothelial function and ameliorates insulin sensitivity and inflammation in cardiopathic nondiabetic patients after an aortocoronary bypass. Metabolism. 2009, 58(9):1270-6	Included	--
Martina et al. Long-term N-acetylcysteine and L-arginine administration reduces endothelial activation and systolic blood pressure in hypertensive patients with type 2 diabetes. Diabetes Care. 2008, 31(5):940-4.	Included	--
Wilson et al. Circulation. L-Arginine Supplementation in Peripheral Arterial Disease: No Benefit and Possible Harmful. 2007. 10;116(2):188-95.	Included	--
Maxwell et al. 2002. Randomized trial of a medical food for the dietary management of chronic, stable angina. J Am Coll Cardiol.Jan 2;39(1):37-45	Included	--
Walker et al. Endothelium-dependent vasodilation is independent of the plasma L-arginine/ADMA ratio in men with stable angina: lack of effect of oral L-arginine on endothelial function, oxidative stress and exercise performance. J Am Coll Cardiol. 2001, 38(2): 499-505.	Included	--
Blum et al. Oral L-arginine in patients with coronary artery disease on medical management. Circulation. 2000, 101(18): 2160-4.	Included	-
Adams et al. Oral L-arginine improves endothelium-dependent dilatation and reduces monocyte adhesion to endothelial cells in young men with coronary artery disease. Atherosclerosis. 1997, 129(2):261-9.	Included	--

Rector et al. Randomized, double-blind, placebo-controlled study of supplemental oral L-arginine in patients with heart failure. Circulation. 1996, 93(12):2135-41.	Included	--
Bahram et al. Effect of l-arginine and selenium on metabolic features, insulin resistance and hepatic function tests in obese women. Current Nutrition & Food Science. 2016: 11(2): 93-98.	Excluded	Criterion of obesity other than the eligible (BMI)
Deveaux et al. L-Arginine supplementation alleviates postprandial endothelial dysfunction when baseline fasting plasma arginine concentration is low: a randomized controlled trial in healthy overweight adults with cardiometabolic risk factors. J Nutr. 2016;146:1330–40.	Excluded	Without eligible population
Kayacelebi et al. Biosynthesis of homoarginine (hArg) and asymmetric dimethylarginine (ADMA) from acutely and chronically administered free L-arginine in humans. Amino Acids. 2015. 47(9):1893-908.	Excluded	Study in duplicate (Schneider et al., 2015)
Dashtabi et al. Oral L-Arginine administration improves anthropometric and biochemical indices associated with cardiovascular diseases in obese patients: a randomized, single blind placebo controlled clinical trial. Res Cardiovasc Med.2015,5(1): 294-319	Excluded	Without eligible outcome
Nascimento et al.ESPEN Journal. Effects of short-term l-arginine supplementation on lipid profile and inflammatory proteins after acute resistance exercise in overweight men. 2014, 9(3): 141– 145	Excluded	Without eligible population and outcome
Suliburska et al. Changes in mineral status are associated with improvements in insulin sensitivity in obese patients following L-arginine supplementation. Eur J Nutr.2014, 53(2):387-93.	Included	Without eligible outcome
Monti et al. L-arginine enriched biscuits improve endothelial function and glucose metabolism: a pilot study in healthy subjects and a cross-over study in subjects with impaired glucose tolerance and metabolic syndrome. Metabolism. 2013, 62(2): 255-64.	Included	Without eligible type of intervention
Bogdanski et al. Effect of 3-month L-arginine supplementation on insulin resistance and tumor necrosis factor activity in patients with visceral obesity. Eur Rev Med Pharmacol Sci. 2012, 16(6): 816-23.	Included	Without eligible outcome
Figueroa et al. Effects of watermelon supplementation on arterial stiffness and wave reflection amplitude in postmenopausal women. Menopause. 2013. 20(5): 573-7.	Excluded	Without eligible intervention
Tripathi et al. Role of L-Arginine on dyslipidemic Conditions of Acute Myocardial Infarction Patients. Indian J Clin Biochem. 2012, 27(3): 296-9.	Excluded	Without eligible design of study
Jabłecka et al. The effect of oral L-arginine supplementation on fasting glucose, HbA1c, nitric oxide and total antioxidant status in diabetic patients with atherosclerotic peripheral arterial disease of lower extremities. Eur Rev Med Pharmacol Sci. 2012, 16(3): 342-50	Excluded	Without eligible comparator
Jude et al. Effect of L-arginine on the microcirculation in the neuropathic diabetic foot in Type 2 diabetes mellitus: a double-blind, placebo-controlled study. Diabet Med. 2010, 27(1): 113-6	Excluded	Lack of author´s response

Orea-Tejeda et al. The effect of L-arginine and citrulline on endothelial function in patients in heart failure with preserved ejection fraction. Cardiol J. 2010. 17(5): 464-70	Excluded	Without eligible comparator
Siasos et al. The impact of oral L-arginine supplementation on acute smoking-induced endothelial injury and arterialperformance. Am J Hypertens. 2009, 22(6): 586-92. 10.1038/ajh.2009.57.	Excluded	Without eligible population
Siasos et al. Short-term treatment with L-arginine prevents the smoking-induced impairment of endothelial function and vascular elastic properties in young individuals Int J Cardiol. 2008, 126(3):394-9.	Excluded	Without eligible population
Schwedhelm et al. Pharmacokinetic and pharmacodynamic properties of oral L-citrulline and L-arginine: impact on nitric oxide metabolism Br J Clin Pharmacol. 2007, 65(1):51-9.	Excluded	Without eligible population
Böger et al. Asymmetric dimethylarginine determines the improvement of endothelium-dependent vasodilation by simvastatin: Effect of combination with oral L-arginine. J Am Coll Cardiol. 2007, 12;49(23): 2274-82.	Excluded	Without eligible population and comparator
Doutreleau et al. Chronic L-arginine supplementation enhances endurance exercise tolerance in heart Int J Sports Med. 2006, 27(7): 567-72.	Excluded	Without eligible design of study
Lucotti et al. Beneficial effects of a long-term oral L-arginine treatment added to a hypocaloric diet and exercise training program in obese, insulin-resistant type 2 diabetic patients. Am J Physiol Endocrinol Metab. 2006, 291(5): 906-12	Included	Without eligible outcome
Schulman et al. L-arginine therapy in acute myocardial infarction: the Vascular Interaction With Age in Myocardial Infarction (VINTAGE MI) randomized clinical trial. JAMA. 2006, 4 (1):58-64.	Excluded	Different measurement technique for analysis of outcome (blood flow)
Yin et al. L-arginine improves endothelial function and reduces LDL oxidation in patients with stable coronary artery disease. Clin Nutr. 2005: 24(6): 988-97.	Excluded	Without eligible comparator
Bednarz et al. L-arginine supplementation prolongs exercise capacity in congestive heart failure. Kardiol Pol. 2004, 60(4): 348-53.	Excluded	Full text in Polish
Dudek et al. L-arginine supplementation does not inhibit neointimal formation after coronary stenting in human beings: an intravascular ultrasound study. Am Heart J. 2004, 147(4):E12	Excluded	Without eligible comparator
Regensteiner et al. Oral L-arginine and vitamins E and C improve endothelial function in women with type 2 diabetes. Vasc Med. 2003, 8(3): 169-75.	Excluded	Without eligible comparator
Bode-Böger et al. Oral L-arginine improves endothelial function in healthy individuals older than 70 years Vasc Med. 2003, 8(2): 77-81.	Excluded	Without eligible population
Abdelhamed et al. No effect of an L-arginine-enriched medical food Am Heart J. 2003, 145(3):15.	Excluded	Without eligible population

Miller et al. Effects of an acute dose of L-arginine during coronary angiography in patients with chronic renal failure: a randomized, parallel, double-blind clinical trial. Am J Nephrol.2003, 23(2):91-5.	Excluded	Without eligible intervention
Huynh et al. Oral arginine reduces systemic blood pressure in type 2 diabetes: its potential role in nitric oxide generation. J Am Coll Nutr. 2002, 21(5):422-7.	Excluded	Without eligible comparator
Sozykin et al. Effect of L-arginine on platelet aggregation, endothelial function adn exercise tolerance in patients with stable angina pectoris. Ter Arkh. 2000, 72(8):24-7	Excluded	Full text in Russian
Hambrecht et al. Correction of endothelial dysfunction in chronic heart failure: additional. J Am Coll Cardiol. 2000, 35(3):706-13.	Excluded	Without eligible comparator
Piatti et al. Long-term oral L-arginine administration improves peripheral and hepatic insulin sensitivity in type 2 diabetic patients. Diabetes Care. 2001, 24(5):875-80.	Excluded	Without eligible outcome
Blum et al. Clinical and inflammatory effects of dietary L-arginine in patients with intractable angina pectoris. Am J Cardiol. 1999, 83(10):1488-90.	Excluded	Without eligible design of study
Bellamy et al. Syndrome X and endothelial dysfunction. Cardiovasc Res. 1998. 40(2):410-7.	Excluded	Without eligible population
Lerman et al. Long-term L-arginine supplementation improves small-vessel coronary endothelial function in humans. Circulation. 1998. 97(21):2123-8.	Excluded	Without eligible population
Contreras et al. Effects of aspirin or basic amino acids on collagen cross-links and complications in NIDDM. Diabetes Care. 1997, 20(5):832-5.	Excluded	Without eligible outcome
Ceremuzyński et al. Effect of supplemental oral L-arginine on exercise capacity in patients with stable angina pectoris. American Journal of Cardiology. 1997, 80(3): 331-333.	Excluded	Without eligible outcome
Khan et al. Oral L-arginine supplementation and cutaneous vascular responses in patients with primary Raynaud's phenomenon. Arthritis and Rheumatism. 1997, 40(2): 352-357.	Excluded	Without eligible population
Chin-Dusting et al. Dietary supplementation with L-arginine fails to restore endothelial function in forearm resistance arteries of patients with severe heart failure. J Am Coll Cardiol. 1996, 27(5):1207-13.	Included	Lack of author´s response
Clarkson et al. Oral L-arginine improves endothelium-dependent dilation in hypercholesterolemic young adults.J Clin Invest. 1996, 97(8):1989-94.	Excluded	Without eligible population
Adams et al. Oral L-arginine inhibits platelet aggregation but does not enhance endothelium-dependent dilation in healthy young men. J Am Coll Cardiol. 1995, 26(4):1054-61.	Excluded	Without eligible population
Gater et al. Effects of arginine/lysine supplementation and resistance training on glucose tolerance. J Appl Physiol. 1992, 72(4):1279-84	Excluded	Without eligible population
